# Culinary strategies to manage glycemic response in people with type 2 diabetes: A narrative review

**DOI:** 10.3389/fnut.2022.1025993

**Published:** 2022-11-10

**Authors:** Serafin Murillo, Ariadna Mallol, Alba Adot, Fabiola Juárez, Alba Coll, Isabella Gastaldo, Elena Roura

**Affiliations:** ^1^Health and Food Habits Department, Fundació Alicia, Sant Fruitós de Bages, Spain; ^2^Endocrinology and Nutrition Department, Universitat de Barcelona, Barcelona, Spain; ^3^Endocrinology and Nutrition Department, Hospital Sant Joan de Déu, Esplugues de Llobregat, Spain

**Keywords:** type 2 diabetes, diabetes management, glycemic, glycemic index, culinary strategies, cooking techniques

## Abstract

Diet plays a critical role in the management of many chronic diseases. It is well known that individuals with type 2 diabetes (T2D) need to pay close attention to foods rich in carbohydrates to better manage their blood sugar. Usually, individuals are told to increase their dietary fiber intake which is associated with better glycemic control and limit their overall carbohydrate consumption. However, there are many other cooking strategies available to reduce the glycemic response to meals rich in carbohydrates and with a high glycemic index, such as adding fats, proteins, or vinegar, modifying the cooking or preparation processes, and even the selection and storage of foods consumed. The aim of the present narrative review is to summarize some of these existing strategies applied to the cooking process and their ability to modulate glycemic response to meals in individuals with T2D.

## Introduction

The increase in the global prevalence of type 2 diabetes (T2D) and associated chronic diseases has prompted a lot of research into possible strategies to reduce its impact on society (1–3). Healthy eating is an essential component of treatment, and it is fundamental to identify and implement effective dietary strategies to prevent and treat the progression of the disease (1).

For people with T2D, nutritional goals should be the same as for the rest of the population: a healthy, balanced diet, adapted to each individual and incorporating foods from all groups (3). In addition to that, patients with T2D need to pay special attention to foods rich in carbohydrates that may be included in each meal, to avoid rapid changes in blood glucose.

According to the American Diabetes Association (4), the total amount of carbohydrates in a meal is the most important determinant of postprandial glycemic response. However, the quality of carbohydrate-rich foods may have additional benefits on post-meal blood glucose levels. One of the most common and usual ways of measuring the glycemic effect of food is by using the glycemic index (GI) (5, 6). This scale ranks a carbohydrate-containing food or drink by how much it raises blood glucose levels, compared with the effect caused by eating the same amount of glucose or white bread as a baseline. On the other hand, the concept of glycemic load (GL) refers to the hyperglycemic effect of a food, in this case, according to the amount of carbohydrates contained in the portion. However, the application of GI and GL concepts has important limitations, since there is a high intra- and interindividual variability in the glycemic response (7). One of the reasons is the high variability of dietary and cooking factors that can modify the glycemic index of foods, therefore influencing the glycemic response (GR). These include the combination of foods with different nutritional compositions, to handling techniques, and cooking processes. In the case of T2D, those that minimize GR are noteworthy (1, 8–14).

This narrative review collects the evidence published to date on the ability of certain dietary and cooking strategies to reduce the GR of foods rich in carbohydrates. The objective is to review the magnitude of the impact that they can have and to reveal those that can be carried out in a simple and practical way on a day-to-day basis, both to help health care professionals when giving recommendations, and people with T2D.

## Methods

The methodology of the current narrative review was based on searching the PubMed and Cochrane Library databases. The search was conducted from March 2021 to December 2021 with the use of different keywords related to diabetes and combinations, taking different spelling of search terms into consideration such as: (“glycemic/glycaemic index” OR “glycemic/glycaemic load” OR “glycemic/glycaemic response” OR “glycemic/glycaemic variability” OR “blood glucose response” OR “glucose absorption” OR “postprandial glycemia”) AND (“cooking” OR “culinary techniques” OR “cooling” OR “resistant starch” OR “retrogradation” OR “diet therapy” OR “dietary strategies”) AND (“type 2 diabetes mellitus” OR “diabetes mellitus” OR “diabetes management” OR “insulin resistance” OR “insulin sensitivity” OR “antidiabetic activity”), adding relevant cited sources and cross-references. Subsequently, titles, abstracts and finally full-text articles were examined by the scientific team about the suitability of the articles in terms of content and, in a subsequent step, in terms of quality. For the purposes of this review, studies included were limited to those published in English, conducted in healthy or individuals with T2D and carried out in the last 30 years.

## Results

### Combination of foods rich in carbohydrates and foods with different nutritional profiles

It is known that the GR of a food can be influenced by the simultaneous intake of other foods with a different nutritional profile (15–17). Specifically, the presence or combination of foods rich in fiber, fat and/or protein, or the addition of vinegar to foods rich in carbohydrates delays the so-called gastric emptying (15, 16, 18). That is, the combination of foods rich in carbohydrates with foods with a different nutritional profile (each with its corresponding absorption speed), slows down the time it takes for food to pass from the stomach to the intestine, slowing down the absorption of food and giving a lower GR. Below, is a detailed description of how the combination of foods rich in carbohydrates with foods with a different nutritional profile (fiber, fat, protein, and acetic acid) can particularly affect GR.

#### Fiber

Dietary fiber is a type of carbohydrate of plant origin present in fruits, vegetables, legumes, seeds, nuts and whole grains resistant to the action of digestive enzymes, without being able to be digested by the body and, therefore, without direct influence on the GR (19, 20). It reaches the large intestine intact, where the intestinal bacteria will be able to metabolize, generating short-chain fatty acids (butyrate, propionate, and acetate) that are beneficial for the body (18, 20, 21). The higher fiber content of a meal can help lowering the GI of a food.

In previous research it was observed that the GR of a food with a high GI can be reduced if it is combined with foods rich in fiber (12, 22), especially those rich in soluble fiber (21). Comparing a breakfast with a high GI (2.2 ± 0.2g fiber) to another with a low GI (4.9 ± 0.5g fiber), a 46% decrease in GR was observed in the latter, measured as the area under the curve (AUC) (22). In the study by Guzman G, et al., where they compared the GR generated after the consumption of fruit juice with and without pulp, and from an entire piece of fruit, those who ate the entire fruit presented a significant decrease in AUC compared to those who consumed fruit juice with and without pulp (689 ± 70.7 mg/dl, 892 ± 70.7 mg/dl, 974 ± 70.7 mg/dl, respectively) (23).

Another study based on people with T2D, it was compared the effects of eating the same amount of carbohydrates in the form of white rice or in the form of white rice mixed with beans or chickpeas (foods rich in fiber) on blood glucose. The combination of rice with beans or chickpeas was shown to attenuate GR compared to rice alone (24).

In conclusion, the consumption of foods rich in carbohydrates with a high GI, together with foods rich in fiber (fruits and vegetables, whole grains, legumes, and nuts) attenuates GR.

#### Fat

Evidence in type 1 diabetes individuals suggests that meals rich in carbohydrates and fats cause a progressive and sustained increase in GR over time (25). By adding foods rich in fat to dishes, gastric emptying is slowed down, reducing the digestion of carbohydrates in the intestine and, therefore, the GR (17, 26–28).

According to some studies carried out with type 2 people with diabetes, the addition of fat is related to a greater stability of glucose levels and a reduction in the GR (17, 29).

The Liljeberg study assessed the impact of adding different high-fat sauces (tomato sauce with extra virgin olive oil or pesto), to a rice dish and a pasta dish. White rice showed an increase in AUC compared to pasta (2,236.84 ± 217.92 mg min/dl and 1,478.62 ± 232.33 mg min/dl, respectively) that was reduced depending on the sauce used (rice + tomato sauce with olive oil = 1,509.24 ± 185.5 mg min/dl; rice + pesto = 1,395.78 ± 87.3 mg min/dl). Adding these sauces on pasta dishes, it was observed a major reduction by adding tomato sauce compared to pesto (pasta + tomato sauce with olive oil = 920.31 ± 124.27 mg min/dl; pasta + pesto = 1,055.39 ± 189.11 mg min/dl) (17). Similar results were obtained in another study where they observed the GR presented by three high GI foods (pasta, potato and toast) when adding cheddar cheese (120 g with 42 g of fat). When combining pasta with cheddar cheese, a reduction in AUC of 58% was observed, 56% when combined with potato and 30% when combined with toast compared to the consumption of their respective products without cheese (30).

In conclusion, the consumption of foods rich in carbohydrates together with foods rich in fat (extra virgin olive oil, oily fish, nuts, and seeds) attenuates the GR.

#### Protein

Apart from delaying gastric emptying, slowing down absorption and reducing GR, proteins are capable of interacting with the starch granules present in starchy foods, limiting the accessibility of enzymes and hindering digestion and subsequent absorption (31). In addition, they increase insulin secretion, decreasing the GR (28).

Current evidence indicates that a slight increase in protein content (25% of total calories) in the diet of healthy people helps to reduce the GR of carbohydrate-rich foods (18, 24, 32). Similar results have been seen in people with diabetes, showing that a high-protein breakfast (30 g protein) lowers glucose levels 180 min after eating (124 ± 3 mg/dl) compared to those low in protein (10 and 20 g of protein; 143 ± 3 mg/dl and 125 ± 3 mg/dl, respectively) and generally improves glucose control throughout the day (33).

A study compared the GR of the same amount of rice consumed alone or in combination with 25 g of protein from different protein sources such as soy, chicken, fish and egg in healthy participants as well as people with diabetes. Among the different protein sources, a hierarchy was perceived, with soya beancurd being the protein source that significantly reduced GR, followed by chicken, fish and egg (34, 35). In the study by Henry, C et al., the effect of tuna was observed in combination with a high GI food such as potato and pasta, showing a reduction in GR of 18 and 54%, respectively (15).

In short, the consumption of foods rich in carbohydrate together with foods rich in protein of animal origin (meat, fish, eggs, dairy products, and derivatives) or vegetal origin (legumes, nuts, and seeds) attenuates GR. However, more studies are still needed in this regard.

#### Acetic acid (vinegar)

Condiments and seasonings are also part of a healthy diet and characteristic of each culture. Recently, it has been studied whether they can contribute to reducing the GR of our dishes. One of the most studied condiments has been vinegar.

Acidity is a limiting factor against the speed of gastric emptying, thus being reduced in foods with a high GI (18). In a study with people with T2D, the effect of adding vinegar to a meal with a high GI compared to another with a low GI was observed. The group that ate the meal with a high GI + vinegar had a lower AUC (3,259.81 ± 1,404.78 mg min/dl) compared to those that did not add vinegar to said meal (5,601.11 ± 2,233.24 mg min/dl). In contrast, no significant differences were observed in the group that consumed a meal with a low GI + vinegar (4,124.29 ± 684.38 mg min/dl) compared to the same meal without adding vinegar (4,286.38 ± 450.25 mg min/dl) (36). In the study by Ostman et al., a 35% decrease in GI was observed when 18 mmol acetic acid (equivalent to 20 g of vinegar) was combined with high GI foods, such as white bread (37). Meanwhile, Liljeberg et al. observed an 11% decrease in the GI when the vinegar was administered in the form of a vinaigrette, including water and olive oil (8 g), a fact that may help explain this more significant drop in the glycemic response (37, 38).

In other recent studies, the consumption of vinegar (15–20 ml/day) before or in combination with high GI foods was associated with an improvement in GR and insulin sensitivity (39, 40). Similar effects were also observed when consuming pickled foods such as pickles (27, 28, 37). According to Leeman et al., refrigerating starchy foods such as boiled potatoes and the subsequent addition of vinegar decreased their GI by up to 43% (41).

These properties have been attributed to acetic acid, which is capable of slowing down stomach emptying by inhibiting digestive enzymes (39, 40, 42). This way, the speed at which carbohydrates are digested is reduced, causing the GR to be lower (43). Based on available evidence, Santos hypothesized three pathways by which vinegar may improve blood glucose: the inhibition of α-amylase, increased glucose uptake and mediation by transcription factors (42). However, to understand mechanisms underlying the antihyperglycemic effects of acetic acid, further experiments are still required. Similar effects have been observed with the addition of lactic acid to starchy foods (31). Therefore, fermentation or food processing that incorporates organic acids has beneficial effects on glucose metabolism, reducing the GR (28, 31, 39, 43).

Overall, the addition of vinegar or pickled foods to meals could be an interesting recommendation for people with T2D, although more studies are still necessary to evaluate the optimal amount to add to foods rich in carbohydrates to observe a significant reduction in GR.

### Food processes and preparation

There are different processes to which foods are subjected to during the culinary course: from the ripening process of the food itself, to its processing, cooking, and conservation, which can all have an impact on GR. Some of these processes, such as milling, pressing, and extrusion can modify size particles of foods rich in carbohydrates, affecting digestion, absorption, and consequently, the GR (14, 44). In addition, when starches present in certain foods rich in carbohydrates are cooked, they are modified, directly affecting digestibility and GR (27). Another key factor is the storage of foods rich in starches, a process in which the generation of resistant starch may take place. Foods containing resistant starch (RS) generally have a lower GR since it is not digested in the small intestine, reaching the large intestine where it is fermented by intestinal bacteria (45).

Different types of starches are distinguished according to their structure, which will determine the response to digestion (for a summary, see Table 1). Type 1 starch (RS1) is one that is physically inaccessible because it is covered by a food matrix, making it difficult for it to interact with digestive enzymes. Regardless of the matrix, some starch granules (without any prior treatment) appear to be naturally resistant to digestion (RS2). Starch that is cooked and refrigerated, losing its structure, and becoming less accessible, is known as type 3 or retrograde starch (RS3). Lastly, we have the starches that have been chemically modified (RS4) (45–47).

**TABLE 1:** Classification of types of resistant starch (RS) and food sources [adapted from Raigond, et al. (46)].

Type of resistant starch	Description	Food sources
RS1	Physically inaccessible, non-digestible matrix	Whole or partly milled grains and seeds
RS2	Ungelatinized resistant starch granules	Raw potato starch, green bananas, high-amylose corn starch
RS3	Retrograded starch (cooled gelatinized starch)	Cooked and cooled potato, bread, pasta, rice, food products with repeated moist heat treatment
RS4	Chemically modified starch	Etherized, esterified or cross-bonded starches (used in processed foods: breads, cakes)

Each of the different modifications that foods can undergo and their impact on GR are detailed below.

#### Level of ripeness of fruits rich in starch

During the ripening process, fruits undergo a series of organoleptic and physical changes. Even its nutritional composition can vary. In general, the green color of fruits decreases due to the loss of chlorophyll while other pigment concentrations like carotenoids remain fairly constant. The color of the fruits depends on the concentration of the unmasked pigments. The texture of the fruits changes due to the hydrolysis of the complex carbohydrates (starches) and other components present, due to the reduction of their fiber content and due to the degradation of the cell walls. With the hydrolysis of starches, sugars are formed, thus providing a sweeter taste. Also, it is observed a change in flavor and aroma due to the presence of esters, organic acids and other components, produced during ripening more flavor and aroma (48–51).

In the earliest stages, the starch content is higher, being resistant to digestion, specifically to the alpha-amylase enzyme, and reaching the colon intact, where it will be fermented by the microbiota. Thus, much more time is required compared to the absorption of simple sugars already present in the later phases, making underripe fruits a good source of carbohydrates and a good alternative to reduce GR in people with T2D (51).

During the ripening process of fruits, highlighting climacteric fruits, those that after being harvested continue with the ripening process (banana, apple, pear, kiwi, peach, melon, plum or fig, among others), the starch is converted to simple sugars (fructose and glucose), thus increasing its GI and, therefore, its RG (49, 52).

There are some studies that assessed the effect of the degree of ripening of fruits on blood glucose. In any case, the variety of fruits studied was not very wide, focusing mainly on bananas (49, 51).

As observed by Hermansen et al. green bananas have a higher starch content (80-90%) and thus a lower GR (1,116.62 ± 306.17 mg/dl) compared to ripe bananas (1,909.06 ± 306.17 mg/dl) which have higher amounts of free sugars (49).

In short, it is interesting to take into account the degree of maturation of fruits rich in starch, opting for the less mature versions that have a higher content of fiber and resistant starch.

#### Degree of processing (integrity and structure)

Food integrity and structure also influence GR in people with T2D (14, 44, 53–55). Those foods that preserve their original structure, without being subjected to any processing technique (27, 56), expose their nutrients within the food matrix. During chewing and digestion, their structure is broken, and different nutrients are absorbed. These foods require more energy to break down, and this process takes longer, with post-prandial glycemia being reduced (56–58). It should also be remembered that in this type of process, part of the fiber originally contained can be eliminated in the food, which would affect the GR.

Some studies have shown the importance of maintaining food structure in order to reduce GR (53–55). Comparing the size particles of foods such as oats (flour vs. flakes), an increase in blood glucose was observed after consuming the flour due to a greater availability of starch compared to whole flakes. The latter delays the absorption of starch, seeing GR significantly reduced (55). In fact, in another study looked at the GI of whole guava (29 ± 4), whole papaya (38 ± 2), guava puree (47 ± 4) and papaya puree (42.5 ± 5) demonstrating that the pureed versions had a higher GI (59).

The food matrix interacts with nutrients, obtaining different outcomes when these nutrients and bioactive compounds are isolated. This has a significant impact on nutrient absorption and a subsequent effect on blood glucose (60, 61). The food matrix protects nutrients by making them less accessible to the digestion process, a fact that leads to a gradual rise in blood glucose levels (31). Thus, being a good option for people with T2D to avoid exaggerated changes in glucose levels and overstimulation of the pancreas.

To summarize, any processing technique that modifies the food’s original structure breaks down the internal components of the food, exposing them to digestion, facilitating absorption, and altering blood glucose. It is therefore, important to consume foods that preserve their structure and integrity, prioritizing whole foods and not their processed varieties.

#### Cooking

The cooking of food destabilizes starch granules allowing glucose chains to form gels (gelatinization), making their digestion and subsequent absorption easier. However, different cooking techniques result in different outcomes. Comparing the resistant starch generated when cooking rice noodles with different techniques (boiled, steamed, microwaved, sautéed, and fried), it was observed that microwave cooking resulted in the highest content of resistant starch (0.99 g/100 g), a substantially higher content compared to the rest of the cooking methods analyzed: sautéed noodles (0.59 g/100 g), steamed noodles (0.44 g/100 g), and boiled noodles (0.43 g/100 g). Therefore, microwave cooking resulted in a higher content of resistant starch and, therefore, its consumption would cause a lower GR compared to the rest of the cooking techniques analyzed (27, 62). However, the amount of resistant starch generated by cooking is less than when using post-cooking refrigeration methods (see section “Storage”).

In addition to cooking methods, cooking time is another factor to consider. Recent studies show that cooking foods for a longer time increases the GR. Therefore, long cooking times favor digestion and increase GR, while short cooking times, such as pasta cooked al dente, will lead to a more controlled rise in glucose levels (31, 63).

The amount of liquid in which foods rich in starches are cooked in also influences the GR. The studies by Kaur et al. and Wu et al. observed that a 2:1 water-rice ratio significantly increases the content of resistant starch in white rice compared to a 3:1 or 4:1 ratio (27, 64), with the first being the best option to avoid large alterations in glycemia.

In summary, it is interesting to carry out shorter cooking times (al dente) and reduce the amount of cooking water, to increase the concentration of resistant starch, decreasing the GR.

#### Storage

**FIGURE 1:**
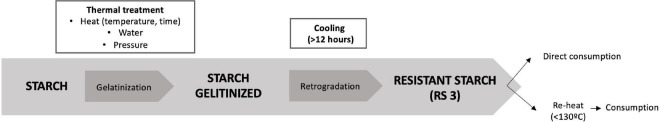
Resistant starch formation. When applying heat treatment, or cooking to a food rich in starch, it becomes gelatinized. If it is subsequently cooled for a minimum of 12 h, it will retrograde, thus obtaining type 3 resistant starch. The food can be consumed directly or reheated to a temperature of <130°C, preventing it from turning back into the non-resistant structure.

The temperature and storage time of foods rich in starch cause changes in its molecular structure, having a different impact on blood sugar (65). If, following cooking, the starch is allowed to cool (retrogradation), the starch granules shrink due to loss of water and the particles reorganize themselves into a crystalline structure, known as resistant starch type 3 (RS3), which offers a much more complex digestion, remaining intact throughout the intestinal tract, and acting as a prebiotic (Figure 1) (12, 27, 45, 46, 66).

Given that storage conditions and conservation factors have the ability to modify contents of RS3 (67), some studies have assessed the impact that storage of foods rich in starches have on GI (12, 57). The content of resistant starch in foods such as potatoes was analyzed under specific temperature conditions: cooking at 65°C (0.64 g/100 g), refrigerated at 4°C for 24 h (1.30 g/100 g) and refrigerated but later reheated (1.65 g/100 g). The foods which showed a higher content of resistant starch were those baked and after 24 h of refrigeration, reheated (12, 57). According to existing evidence, resistant starch is not turned back into its non-resistant structure when reheating temperatures do not exceed 130°C (12).

Hsin Yang et al. evaluated the GR of white rice cooked and stored at different temperatures (4, 0, and −20°C), and with a control sample stored at 25°C. In addition to that, these boiled rice samples were stored and analyzed over different days (day 1, 3, and 5). They found that the rice stored at 0°C had a lower GR, measured by AUC (2,032 ± 191 mg/dl), followed by the sample stored at 4°C (2,032 ± 193 mg/dl), −20°C (2,111 ± 182 mg/dl) and finally, 25°C (2,258 ± 191 mg/dl). Regarding the days of storage, the sample that was kept for 5 days at 0°C, presented a lower AUC (1,806 ± 182 mg/d°) than those samples stored for 1 and 3 days (2,032 ± 191 mg/dl, 1,881 ± 184 mg/dl, respectively) (68).

Any process that decreases the moisture content of starchy foods during storage or preparation also influences the formation of gels, altering their absorption. A study evaluated the impact of freezing and toasting bread on GR, and found that both techniques modified the GR of bread, but highlighted that bread that had been frozen, thawed and then re-toasted had a lower GR (2,827.57 mg min/dl) compared to bread frozen and thawed, directly toasted, or fresh (3,223.79, 3,475.93, and 4,664.59 mg min/dl, respectively) (57).

In conclusion, those strategies that reduce the water content of foods rich in starch are interesting, such as, for example, toasting in addition to refrigerating the food after cooking to generate resistant starch, and in turn reduce the GR of the food.

### Consumption order

In addition to meal composition and size, researchers have also looked at the impact of different foods on GR when eaten in a specific order (58). Recently, it has been seen that consuming protein, fat and fiber from vegetables before consuming starchy foods reduces postprandial blood glucose by up to 73% and circulating insulin levels by 48% (58, 64, 69, 70), compared to consuming along with, or after eating starchy foods. In fact, adapting the order of food consumption, such as consuming foods rich in fiber, fat, and protein before consuming starchy foods, has been proposed as a novel strategy for the prevention and management of postprandial hyperglycemia (64).

**FIGURE 2:**
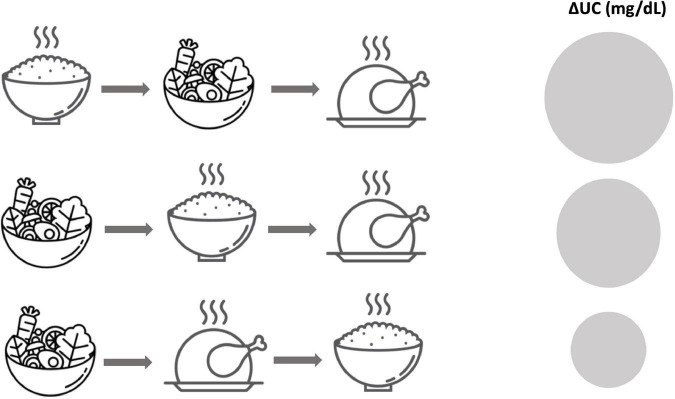
The order of consumption of different food groups has a different impact on the AUC. A significant decrease in AUC (mg/dl) is observed when starchy foods are consumed last, after fiber, fat, and protein. In this figure, adapted from Nishino et al. (58), AUC is represented with a circumference, being larger when the AUC is higher.

In a study with healthy participants, the effects of the same meal consisting of 3 separate components (rice, vegetables, and meat) but in a different order was evaluated (Figure 2). All subjects varied the order of consumption on different days and it was observed that those who consumed rice at the end of the meal did not present a glucose peak of 16.4 ± 5.8 mg/dl until 90 min, compared to the increase observed at 30 min in those who began to eat rice followed by vegetables and meat and those who first consumed vegetables followed by rice and meat (36.0 ± 5.1 and 31.5 ± 5.5 mg/dl, respectively) (58). Similar results were seen in a study with people with diabetes (65). It should be noted that these effects depend on multiple variables such as composition, size and timing of intake, and the degree of individual glucose tolerance (63).

In summary, it is interesting to consider the order of intake to avoid exaggerated rises in blood glucose, prioritizing the consumption of fiber, protein and fat before the intake of starchy foods.

Apart from consumption order, diet can affect the circadian rhythmicity. As commented in Jeyakumar et al. work, from a chronobiological point, glucose metabolism in humans follow a circadian rhythm, through diurnal variation of glucose tolerance that typically peaks during day-light hours, when food consumption usually happens and reduces when it comes to night-dark hours, when fasting usually occurs. Research in this area has suggested that time of day is indicative of having influence on the postprandial glucose response to a meal. In fact, modifying the macronutrient composition of nigh meals, by increasing protein and fat content, has shown to be a simple strategy to improve postprandial glycemia. However, more research is needed to understand the circadian system and its implications on nutrition that may reduce the incidence of T2D (71).

## Conclusion

**FIGURE 3:**
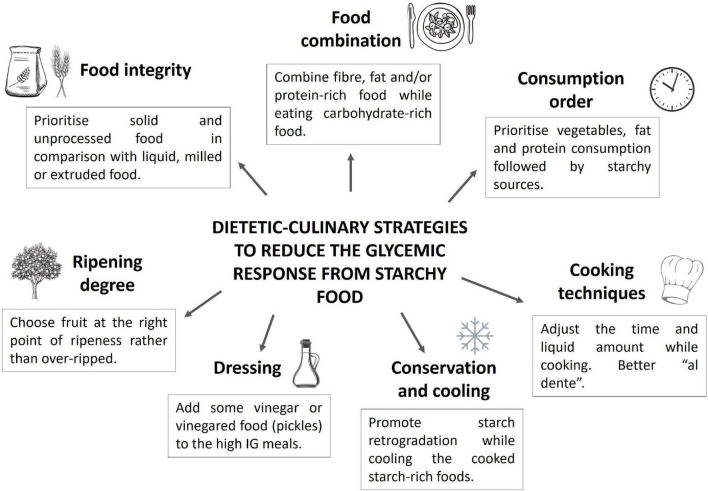
Overview of dietary strategies to reduce glycemic response from starchy foods.

As described in this review, dietary strategies can have an important impact on glycemic control (see Figure 3 for a summary). For this reason, it is important that health care professionals who give advice to patients with diabetes have this knowledge to increase their confidence when it comes to promoting and maintaining health.

This research concludes with some practical and beneficial recommendations to control the GR that are summarized in Table 2.

**TABLE 2:** Practical dietary recommendations for reducing postprandial glycemia in type 2 diabetes (T2D) when starchy foods are consumed.

1. Complement meals rich in carbohydrates with foods rich in fiber (fruits and vegetables, whole grains, legumes, and nuts)
2. Complement meals rich in carbohydrates with foods rich in animal protein (meat, fish, eggs, dairy products, and derivatives) or vegetable protein (legumes, nuts and seeds, and whole grains).
3. Complement meals rich in carbohydrates with foods rich in healthy fats (extra virgin olive oil, oily fish, nuts, and seeds).
4. Add vinegar or incorporate pickled foods to meals rich in carbohydrates with a high GI.
5. Prioritize the consumption of underripe climacteric fruits.
6. Consume foods in its original form, as little processed as possible.
7. Cook pasta, rice or other starchy foods al dente.
8. Prioritize microwave cooking over other cooking techniques (boiling, steaming, sautéing and frying) due to its major content of resistant starch.
9. Cook foods such as pasta, rice or legumes beforehand, leave them to cool in the fridge for 1 day and consume them cold or reheated (<130°C) to allow for the formation of resistant starch.
10. Reduce the proportion of liquid when cooking foods such as rice and pasta.
11. The order matters: preferably consume a first course of vegetables with foods rich in protein and the food rich in carbohydrates as a second course.W3510

## Author contributions

SM and AC: conceptualization. AM: methodology and resources. AM and FJ: formal analysis. IG and AA: research. SM and AM: data curation. AM, FJ, and AA: writing—original draft preparation. SM, ER, IG, and AC: writing—review and editing. SM and ER: supervision. ER: project administration. All authors have read and agreed to the published version of the manuscript.
